# Special Issue “Pediatric Eye Disease: Screening, Causes and Treatment”

**DOI:** 10.3390/children10040654

**Published:** 2023-03-30

**Authors:** Guy L. J. Chen, Jason C. S. Yam, Calvin C. P. Pang

**Affiliations:** Department of Ophthalmology and Visual Sciences, The Chinese University of Hong Kong, Hong Kong, China

Clinical manifestations and courses of eye diseases in children are profoundly variable, from minor irritations, pain, infections, inflammations, ocular misalignment, refractive errors and visual impairment, to permanent blindness [1]. The symptoms and morbid effects of ophthalmic derangements can subside and disappear later in life. In many individuals, however, the morbidities progress irreversibly, and the consequences and burdens are lifelong to them, their families and society as a whole. Concerns for eye health in children are global across different cultures and ethnicities [1,2].

Immediately after birth, the visual system develops steadily. Therefore, infancy and early childhood are exceedingly important in terms of eye health. Congenital pathological conditions, very often genetically determined, such as inherited retinal diseases, congenital glaucoma and congenital cataracts, cannot be prevented. However, other conditions, even when genetics have some etiological roles, such as myopia, can be prevented [3]. Screening for eye diseases in infants or young children is therefore important. Pediatric ocular examinations should be routinely conducted for all infants. In children with high risk of specific diseases, such as those with family history of retinoblastoma or parental history of high myopia, screening for these diseases should be carried out, principally via leukocoria and cycloplegic refraction examination, respectively [1,3].

The prevention of eye diseases in children is crucial and can be made possible. However, the causes of the disease must be known. For example, myopia, essentially developed from childhood, is complex in its etiology. The major form of myopia is caused by elongation of the axial length of the globe. Once developed, it is uncurable and prone to progress. Moreover, serious, blinding complications can occur later in life, including myopic maculopathy, choroidal neovascularization, retinal detachment and glaucoma, especially in subjects with high myopia. Childhood myopia can be prevented by the avoidance of risk factors that occur in daily life, including prolonged close work and few outdoor activities. Optical and pharmacological treatments, especially the administration of low-concentration atropine eye drops [4], are also effective modalities. Over the past several decades, there have been advancements in our understanding of the causes of serious childhood eye diseases such as high myopia and retinoblastoma. However, research is still needed to understand the etiology of diseases such as astigmatism and retinopathy of prematurity.

Early detection is critical for prevention and management. Population screening can detect cases of diseases early. Genetic testing can provide pre-symptomatic diagnoses. Congenital cataracts, which are determined by a single gene and transmitted in autosomal mode, can be effectively treated, and vision can be restored with timely cataract surgery. While treatment protocols are well developed, they still need to be improved for complicated cases. The timing of the cataract surgery is important. Genetic heterogeneity can also have intriguing effects on treatments [5].

Infections and subsequent inflammatory reactions seriously threaten vision in children. Early detection and prompt treatment must be available, and they must also be available for lacrimal dysfunctions, which are not uncommon in some populations.

While some childhood eye diseases are common and have been known for a long time, much work is needed to improve their early diagnoses and treatments for beneficial effects to be seen in the long term. There are also substantial variations in clinical courses of individuals due to genetic and environmental factors. One example is retinopathy of prematurity [6].

In an excellent review, the need for the early detection of eye diseases in childhood and even in infancy for the benefit of effective treatments has been clearly exemplified [7]. A wide range of vision-threatening and blinding diseases require prompt diagnosis and treatment, including retinoblastoma, congenital glaucoma, congenital cataracts, keratoconus, retinopathy of prematurity, strabismus and amblyopia. Modern technologies are being increasingly applied. Smart phones can be used to provide convenient and effective eye screening for children [8,9].

Improvements have been made in the prevention, early detection, diagnosis, treatment and management of pediatric eye diseases over the last few decades. The health burden is still heavy. Enhancements and improvements are still much needed on all fronts.
